# Supplementary material for: A simple and general approach to generate photoactivatable DNA processing enzymes
Source: Nucleic Acids Res. 2021 Dec 14;50(6):e31. doi: 10.1093/nar/gkab1212 (PMC8989547; doi:10.1093/nar/gkab1212)
Supplement: gkab1212_Supplemental_File [file gkab1212_supplemental_file.pdf]

Supporting Information

Oligonucleotide	DNA polymerase-Oligo
ttcctctaccacctacatca*c-DBCO	Phi29 pol-PC_oligo/ Pfu pol-PC_oligo/StuI_PC_oligo
DBCO-t*tcctctaccacctacatcac	Taq pol-PC_oligo
cttcatcacactccatctcc*a-DBCO	Phi29 pol-PC_oligoScr
gcattacgtttggtggaccc*t-DBCO	Phi29 pol-PC_oligo2
ttcctctaccacctacatcac-DBCO	Phi29 pol-oligo

Table S1. Sequence for the DBCO modified oligonucleotides. The asterisk (*) indicates the position of the photocleavable linker. PC BMN was used as photocleavable linker in most of the constructs (Phi29 pol-PC_oligo, Pfu pol-PC_oligo, StuI_PC_oligo, Phi29 pol-PC_oligoScr, and Phi29 pol-PC_oligo2). PC linker was used for Taq pol-PC_oligo. Preliminary tests were conducted using PC linker as photocleavable group for Phi29 pol-PC oligo and Pfu pol-PC_oligo providing efficient cleavage.

	N° cycles	Denaturing	Annealing	Extension
Cyclophilin A	36	15 s at 95 °C	20 s at 50 °C	30s at 72 °C
Bir A gene (Pfu)	39	15 s at 95 °C	20 s at 56 °C	1min:30s at 72 °C
Bir A gene (Taq)	39	15 s at 95 °C	20 s at 63 °C	1min:30s at 72 °C
Bir A fragment	39	15 s at 95 °C	20 s at 59 °C	30s at 72 °C

Table S2. Cycling parameters used for PCR experiments. All PCR experiments start with an initial denaturing step (5 min, 95 °C), followed by the cycling loop, and a final 10 min elongation step at 72 °C. The different cycling parameters are summarized in this table and the primers in Table S3.

	Forward Primer	Reverse Primer
Cyclophilin A	TTCGCCATGGTTAACCCGACCGTTTTCTTCG	GAAGCTCGAGCTGACCGCAGTCCGCGATGG
Bir A gene	TCTACCATGGGCAAGGATAACACCGTGCCACTG	ATTAGCTCGAGTTTTTCTGCACTACGCAGGGA
Bir A fragment	AGAGTGTCGTTAATCAGGG	GCTCAAGTAATAAAGCCC

Table S3. Primers used for PCR

**ssDNA name**	**sequence**
FAM exo 3' activity ssDNA	FAM-tctctctctctcctcttcctatattccgtacttc
FAM exo 3' reverse	FAM- gaagtacggaatataggaagaggagag
Exo 3' mismatch forward	tctctctctaatcgctcttcctatattccgtacttc
Exo 5' taq 4pb gap	ggatgagataggatgaagtacgg
Exo 5' taq template	tctctctctaatcgctcttcctatattccgtacttcatcctatctcatcc
Exo 5' taq fork FAM	ttacttcctaggaagagcgattagagagaga-FAM
FAM StuI BMN-Q535 quencher	FAM-ttcaggcctttag- Q535
FAM Methylated StuI BMN-Q535 quencher	FAM-ttcagg*cCtttag- Q535
StuI reverse	ctaaaggcctgaa

Table S4. Oligonucleotides used in the nuclease activity experiments. FAM stands for 6-carboxyfluorescein. The asterisk in FAM Methylated StuI BMN-Q535 quencher denotes the position of the PTO modification, and the uppercase C the methylated cytosine.

Protein sequences

The position of the 4-Azido-L-phenylalanine incorporation is highlighted.

In the case of the Phi29 pol enzyme, the reference protein sequence used was the NCBI: YP_002004529.1, which lacks the first three amino acids when compared with the sequence used in (29). Nevertheless, the numbering used in this reference is kept here for consistency.

N62D Phi29 pol

```
MPRKMYSCDFETTTKVEDCRVWAYGYMNIEDHSEYKIGNSLDEFMAWVLKVQADLYFHdLKFDGAFIINWLERNGFKWSA
DGLPNTYNTIISRMGQWYMIDICLGYKGKRKIHTVIYDSLKKLPFPVKKIAKDFKLTVLKGDIDYHKERPVGYKITPEEY
AYIKNDIQIIAEALLIQFKQGLDRMTAGSDSLKGFKDIITTKKFKKVFPTLSLGLDKEVRYAYRGGFTWLNDRFKEKEIG
EGMVFDVNSLYPAQMYSRLLPYGEPIVFEGKYVWDEDYPLHIQHIRCEFELKEGYIPTIQIKRSRFYKGNEYLKSSGGEI
ADLWLSNVDLELMKEHYDLYNVEYISGLKFKATTGLFKDFIDKWTYIKTTSEGAIKQLAKLMLNSLYGKFASNPDVTGKV
PYLKENGALGFRLGEEETKDPVYTPMGVFITAWARYTTITAAQACYDRIIYCDTDSIHLTGTEIPDVIKDIVDPKKLGYW
AHESTFKRAKYLRQKTYIQDIYMKEVDGKLVEGSPDDYTDIKFSVKCAGMTDKIKKEVTFENFKVGFSRKMKPKPVQVPG
GVVLVDDTFTIKSGazFGSLEHHHHHH
```

Taq pol

```
MGRGMLPLFEPKGRVLLVDGHHLAYRTFHALKGLTTSRGEPVQAVYGFAKSLLKALKEDGDAVIVVFDAKAPSFRHEAYGGYKAGR
APTPEDFPRQLALIKELVDLLGLARLEVPGYEADDVLASLAKKAEKEGYEVRILTADKDLYQLLSDRIHVLHPEGYLITPAWLWEK
YGLRPDQWADYRALTGDESDNLPGVKGIGEKTARKLLEEWGSLEALLKNLDRLKPAIREKILAHMDDLKLSWDLAKVRTDLPLEVD
FAKRREPDRERLRAFLERLEFGSLLHEFGLLESPKALEEAPWPPPEGAFVGFVLSRKEPMWADLLALAAARGGRVHRAPEPYKALR
DLKEARGLLAKDLSVLALREGLGLPPGDDPMLLAYLLDPSNTTPEGVARRYGGEWTEEAGERAALSERLFANLWGRLEGEERLLWL
YREVERPLSAVLAHMEATGVRLDVAYLRALSLEVAEEIARLEAEVFRLAGHPFNLNSRDQLERVLFDELGLPAIGKTEKTGKRSTS
AAVLEALREAHPIVEKILQYRELTKLKSTYIDPLPDLIHPRTGRLHTRFNQTATATGRLSSSDPNLQNIPVRTPLGQRIRRAFIAE
EGWLLVALDYSQIELRVLAHLSGDENLIRVFQEGRDIHTETASWMFGVPREAVDPLMRRAAKTINFGVLYGMSAHRLSQELAIPYE
EAQAFIERYFQSFPKVRAWIEKTLEEGRRRGYVETLFGRRRYVPDLEARVKSVREAAERMAFNMPVQGTAADLMKLAMVKLFPRLE
EMGARMLLQVHDELVLEAPKERAEAVARLAKEVMEGVYPLAVPLEVEVGIGEDWLSAKESGazFGSLEHHHHHH
```

Pfu pol

```
MGILDVDYITEEGKPVIRLFKKENGKFKIEHDRTFRPYIYALLRDDSKIEEVKKITGERHGKIVRIVDVEKVEKKFLGKPITVWKL
YLEHPQDVPTIREKVREHPAVVDIFEYDIPFAKRYLIDKGLIPMEGEEELKILAFDIETLYHEGEEFGKGPIIMISYADENEAKVI
TWKNIDLPYVEVVSSEREMIKRFLRIIREKDPDIIVTYNGDSFDFPYLAKRAEKLGIKLTIGRDGSEPKMQRIGDMTAVEVKGRIH
FDLYHVITRTINLPTYTLEAVYEAIFGKPKEKVYADEIAKAWESGENLERVAKYSMEDAKATYELGKEFLPMEIQLSRLVGQPLWD
VSRSSTGNLVEWFLLRKAYERNEVAPNKPSEEEYQRRLRESYTGGFVKEPEKGLWENIVYLDFRALYPSIIITHNVSPDTLNLEGC
KNYDIAPQVGHKFCKDIPGFIPSLLGHLLEERQKIKTKMKETQDPIEKILLDYRQKAIKLLANSFYGYYGYAKARWYCKECAESVT
AWGRKYIELVWKELEEKFGFKVLYIDTDGLYATIPGGESEEIKKKALEFVKYINSKLPGLLELEYEGFYKRGFFVTKKRYAVIDEE
GKVITRGLEIVRRDWSEIAKETQARVLETILKHGDVEEAVRIVKEVIQKLANYEIPPEKLAIYEQITRPLHEYKAIGPHVAVAKKL
AAKGVKIKPGMVIGYIVLRGDGPISNRAILAEEYDPKKHKYDAEYYIENQVLPAVLRILEGFGYRKEDLRYQKTRQVGLTSWLNIK
KSSGazFGSLEHHHHHH
```

StuI

```
MGSVSAVEQVFLECERARADGDLIQRVSASDKEYHFQNWVQARIEACRLSYDDPGRNTYPDFRLIHHPEGYEVKGLEFPGREADYD
SNSQVPTGNHGGREVFYVFGRYPKAERGVDEYPVVDLVVCHGSFLNADSEYVHKNKSFRGFGSYGDILVRDRKMYVVPTPFALASG
TAGLATLIVPTEFEPQSDTLVQVGELDRTEVDEVIVSYEFNLQTNEMVTHKAPNLNAGKVHSFRAYRSRGAGDSKPVSLAGGRLSG
azFGSLEHHHHHH
```

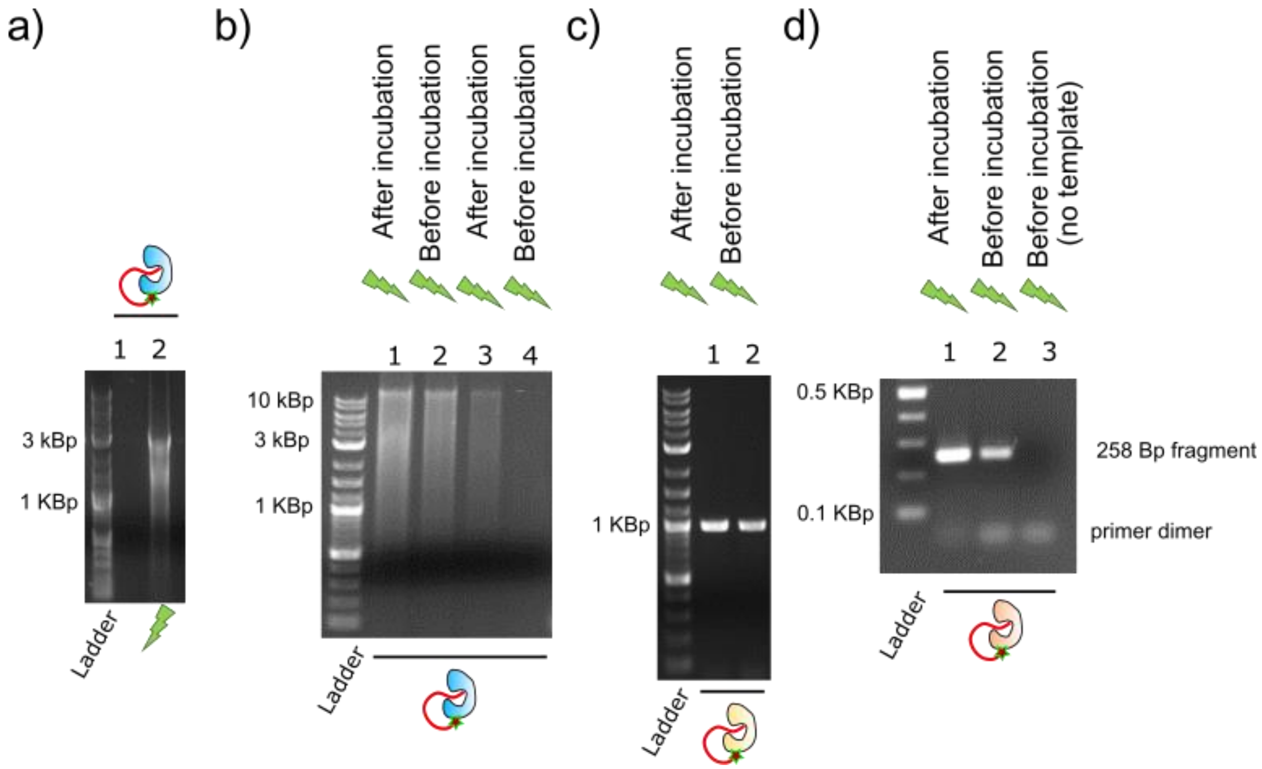

Figure S1.- Tight-blockage of DNA polymerases and further failure-by-design assays. **a)** Tight blockage of the activity of Phi pol-PC_oligo. No amplification product was observed when 150 nM of Phi29 pol-PC_oligo was used (lane 1). The activity was recovered after a 10 s light pulse with 315 nm UV (lane 2). **b), c),** and **d)** Independent failure-by-design experiments were performed to corroborate the results shown in Figure 5. **b)** Whole-genome amplification by Phi pol-PC_oligo. The hexamers concentration in reactions in lanes 1 and 2 was 6.25 µM, and 3.12 µM in lanes 3 and 4. **c)** Light-start PCR amplification of *bir A* gene with Pfu pol-PC_oligo. 10 nM of enzyme was used and 1 µl of the diluted *E. coli* chromosomal DNA sample. **d)** Light-Start with Taq pol-PC_oligo. The same conditions as in Figure 5c were used in this case.

Figure S2 Fidelity of Taq pol–PC_oligo and Pfu pol-PC_oligo enzymes. In order to rule out a significant effect on the fidelity of the amplification reaction, we sequenced PCR products amplified by the oligo-modified enzymes. The *bir A* gene from *E. coli* (GenBank: M15820.1) was PCR amplified, gel-purified, and sent to sequencing (Eurofins Genomics, Germany). The gene was amplified using unmodified Taq pol, unmodified Pfu pol, Tap pol-PC_oligo, and Pfu pol-PC_oligo, and the sequences obtained by the unmodified and oligo-modified versions were compared (a pulse of 120 s 365 nm UV light was used for the activated enzymes). The first 50 and last 100-200 nucleotides of the sequencing reaction were omitted due to limitations of the sequencing reaction. In none of both cases, differences between the sequences retrieved by the unmodified enzymes and the oligo-modified ones were detected (see sequence alignment below). Furthermore, the sequences show 100% identity with the Bir A deposited sequence (GenBank: M15820.1). Altogether, the results are consistent with a conserved fidelity of the light-activated reactions.

Pfu pol *vs* Pfu pol-PC_oligo

```
Alignment of Sequence_1:  [SeqBirA_Pfu.txt.xdna] with  Sequence_2:
[SeqBirA_Pfu_light.txt.xdna]

Similarity : 849/849 (100.00 %)

Seq_1  1     AAGCCCCCTGTTTGTCTATTCCGCGTGAAATGCCAAATATTTCTTTATCACCAATGATAA  60
             ||||||||||||||||||||||||||||||||||||||||||||||||||||||||||||
Seq_2  1     AAGCCCCCTGTTTGTCTATTCCGCGTGAAATGCCAAATATTTCTTTATCACCAATGATAA  60


Seq_1  61    GTTTCACTGGGCGATTAATAAAATTATCCAGCTTTTCCCAGCGCGACAGATAAGGTGCCA  120
             ||||||||||||||||||||||||||||||||||||||||||||||||||||||||||||
Seq_2  61    GTTTCACTGGGCGATTAATAAAATTATCCAGCTTTTCCCAGCGCGACAGATAAGGTGCCA  120


Seq_1  121   ATCCTTCTTGTTCGAAGAGTTCCAACGCAGCACGTAATTCACGTATTAGCATGGCCGCCA  180
             ||||||||||||||||||||||||||||||||||||||||||||||||||||||||||||
Seq_2  121   ATCCTTCTTGTTCGAAGAGTTCCAACGCAGCACGTAATTCACGTATTAGCATGGCCGCCA  180


Seq_1  181   ACGTATTACGATCGAGATTGATCCCCGCTTCCTGCAGCGTGATCCACCCCTGATTAACGA  240
             ||||||||||||||||||||||||||||||||||||||||||||||||||||||||||||
Seq_2  181   ACGTATTACGATCGAGATTGATCCCCGCTTCCTGCAGCGTGATCCACCCCTGATTAACGA  240


Seq_1  241   CACTCTCTTCAACACGGCGCATTGCCATGTTGATCCCGGCTCCAATGACTATTTGCGCCG  300
             ||||||||||||||||||||||||||||||||||||||||||||||||||||||||||||
Seq_2  241   CACTCTCTTCAACACGGCGCATTGCCATGTTGATCCCGGCTCCAATGACTATTTGCGCCG  300


Seq_1  301   CATCGCCAGTTTTGCCAGTCAGCTCCACCAGAATGCCTGCCAGCTTGCGATCCTGCAGAT  360
             ||||||||||||||||||||||||||||||||||||||||||||||||||||||||||||
Seq_2  301   CATCGCCAGTTTTGCCAGTCAGCTCCACCAGAATGCCTGCCAGCTTGCGATCCTGCAGAT  360


Seq_1  361   AGAGGTCATTAGGCCATTTAACACGAACTTTATCTGCACCCAGCTTGCGTAATACTTCCG  420
             ||||||||||||||||||||||||||||||||||||||||||||||||||||||||||||
Seq_2  361   AGAGGTCATTAGGCCATTTAACACGAACTTTATCTGCACCCAGCTTGCGTAATACTTCCG  420


Seq_1  421   CCATCACGATACCGATAACCAGACTTAAACCAATCGCCGCCGCCGGGCCTTGTTCCAGAC  480
             ||||||||||||||||||||||||||||||||||||||||||||||||||||||||||||
Seq_2  421   CCATCACGATACCGATAACCAGACTTAAACCAATCGCCGCCGCCGGGCCTTGTTCCAGAC  480
```

```
Seq_1  481    GCCAGAACATCGACAAATATAAGTTTGCGCCAAAAGGCGAAAACCATTTCCGACCCCGGC  540
              ||||||||||||||||||||||||||||||||||||||||||||||||||||||||||||
Seq_2  481    GCCAGAACATCGACAAATATAAGTTTGCGCCAAAAGGCGAAAACCATTTCCGACCCCGGC  540


Seq_1  541    GACCACGGCCAGCCTGCTGGTATTCTGCAATGCAAGCATCGCCCGATTTAAGCTCTCCGA  600
              ||||||||||||||||||||||||||||||||||||||||||||||||||||||||||||
Seq_2  541    GACCACGGCCAGCCTGCTGGTATTCTGCAATGCAAGCATCGCCCGATTTAAGCTCTCCGA  600


Seq_1  601    TACGATCAAGAAGGTACTGATTCGTGGAGTCAATCACTGGCAGCACGGCTACACTACCGC  660
              ||||||||||||||||||||||||||||||||||||||||||||||||||||||||||||
Seq_2  601    TACGATCAAGAAGGTACTGATTCGTGGAGTCAATCACTGGCAGCACGGCTACACTACCGC  660


Seq_1  661    CATCCAGCTGACCCAATATCTGTTTAGCATTAAGTAACTGGATAGGCTCAGGCAGGCTGT  720
              ||||||||||||||||||||||||||||||||||||||||||||||||||||||||||||
Seq_2  661    CATCCAGCTGACCCAATATCTGTTTAGCATTAAGTAACTGGATAGGCTCAGGCAGGCTGT  720


Seq_1  721    ATCCTTTACCCGGAACGGTAAAGACATCAACGCCCCAGTCACGCAGTGTCTGAATGTGTT  780
              ||||||||||||||||||||||||||||||||||||||||||||||||||||||||||||
Seq_2  721    ATCCTTTACCCGGAACGGTAAAGACATCAACGCCCCAGTCACGCAGTGTCTGAATGTGTT  780


Seq_1  781    TATTAATAGCCGCCCGGCTCATTCCCAGCGTTTCACCCAACTGCTCGCCAGAGTGAAATT  840
              ||||||||||||||||||||||||||||||||||||||||||||||||||||||||||||
Seq_2  781    TATTAATAGCCGCCCGGCTCATTCCCAGCGTTTCACCCAACTGCTCGCCAGAGTGAAATT  840


Seq_1  841    CACCGTTCG  849
              |||||||||
Seq_2  841    CACCGTTCG  849
```

Taq pol vs Taq pol-PC_oligo

Alignment of Sequence_1: [SeqBirA_Taq.txt.xdna] with Sequence_2: [SeqBirA_Taq_light.txt.xdna]

Similarity : 750/750 (100.00 %)

```
Seq_1  1      AAGCCCCCTGTTTGTCTATTCCGCGTGAAATGCCAAATATTTCTTTATCACCAATGATAA  60
              ||||||||||||||||||||||||||||||||||||||||||||||||||||||||||||
Seq_2  1      AAGCCCCCTGTTTGTCTATTCCGCGTGAAATGCCAAATATTTCTTTATCACCAATGATAA  60


Seq_1  61     GTTTCACTGGGCGATTAATAAAATTATCCAGCTTTTCCCAGCGCGACAGATAAGGTGCCA  120
              ||||||||||||||||||||||||||||||||||||||||||||||||||||||||||||
Seq_2  61     GTTTCACTGGGCGATTAATAAAATTATCCAGCTTTTCCCAGCGCGACAGATAAGGTGCCA  120


Seq_1  121    ATCCTTCTTGTTCGAAGAGTTCCAACGCAGCACGTAATTCACGTATTAGCATGGCCGCCA  180
              ||||||||||||||||||||||||||||||||||||||||||||||||||||||||||||
Seq_2  121    ATCCTTCTTGTTCGAAGAGTTCCAACGCAGCACGTAATTCACGTATTAGCATGGCCGCCA  180


Seq_1  181    ACGTATTACGATCGAGATTGATCCCCGCTTCCTGCAGCGTGATCCACCCCTGATTAACGA  240
              ||||||||||||||||||||||||||||||||||||||||||||||||||||||||||||
Seq_2  181    ACGTATTACGATCGAGATTGATCCCCGCTTCCTGCAGCGTGATCCACCCCTGATTAACGA  240
```

```
Seq_1  241   CACTCTCTTCAACACGGCGCATTGCCATGTTGATCCCGGCTCCAATGACTATTTGCGCCG  300
             ||||||||||||||||||||||||||||||||||||||||||||||||||||||||||||
Seq_2  241   CACTCTCTTCAACACGGCGCATTGCCATGTTGATCCCGGCTCCAATGACTATTTGCGCCG  300

Seq_1  301   CATCGCCAGTTTTGCCAGTCAGCTCCACCAGAATGCCTGCCAGCTTGCGATCCTGCAGAT  360
             ||||||||||||||||||||||||||||||||||||||||||||||||||||||||||||
Seq_2  301   CATCGCCAGTTTTGCCAGTCAGCTCCACCAGAATGCCTGCCAGCTTGCGATCCTGCAGAT  360

Seq_1  361   AGAGGTCATTAGGCCATTTAACACGAACTTTATCTGCACCCAGCTTGCGTAATACTTCCG  420
             ||||||||||||||||||||||||||||||||||||||||||||||||||||||||||||
Seq_2  361   AGAGGTCATTAGGCCATTTAACACGAACTTTATCTGCACCCAGCTTGCGTAATACTTCCG  420

Seq_1  421   CCATCACGATACCGATAACCAGACTTAAACCAATCGCCGCCGCCGGGCCTTGTTCCAGAC  480
             ||||||||||||||||||||||||||||||||||||||||||||||||||||||||||||
Seq_2  421   CCATCACGATACCGATAACCAGACTTAAACCAATCGCCGCCGCCGGGCCTTGTTCCAGAC  480

Seq_1  481   GCCAGAACATCGACAAATATAAGTTTGCGCCAAAAGGCGAAAACCATTTCCGACCCCGGC  540
             ||||||||||||||||||||||||||||||||||||||||||||||||||||||||||||
Seq_2  481   GCCAGAACATCGACAAATATAAGTTTGCGCCAAAAGGCGAAAACCATTTCCGACCCCGGC  540

Seq_1  541   GACCACGGCCAGCCTGCTGGTATTCTGCAATGCAAGCATCGCCCGATTTAAGCTCTCCGA  600
             ||||||||||||||||||||||||||||||||||||||||||||||||||||||||||||
Seq_2  541   GACCACGGCCAGCCTGCTGGTATTCTGCAATGCAAGCATCGCCCGATTTAAGCTCTCCGA  600

Seq_1  601   TACGATCAAGAAGGTACTGATTCGTGGAGTCAATCACTGGCAGCACGGCTACACTACCGC  660
             ||||||||||||||||||||||||||||||||||||||||||||||||||||||||||||
Seq_2  601   TACGATCAAGAAGGTACTGATTCGTGGAGTCAATCACTGGCAGCACGGCTACACTACCGC  660

Seq_1  661   CATCCAGCTGACCCAATATCTGTTTAGCATTAAGTAACTGGATAGGCTCAGGCAGGCTGT  720
             ||||||||||||||||||||||||||||||||||||||||||||||||||||||||||||
Seq_2  661   CATCCAGCTGACCCAATATCTGTTTAGCATTAAGTAACTGGATAGGCTCAGGCAGGCTGT  720

Seq_1  721   ATCCTTTACCCGGAACGGTAAAGACATCAA  750
             ||||||||||||||||||||||||||||||
Seq_2  721   ATCCTTTACCCGGAACGGTAAAGACATCAA  750
```

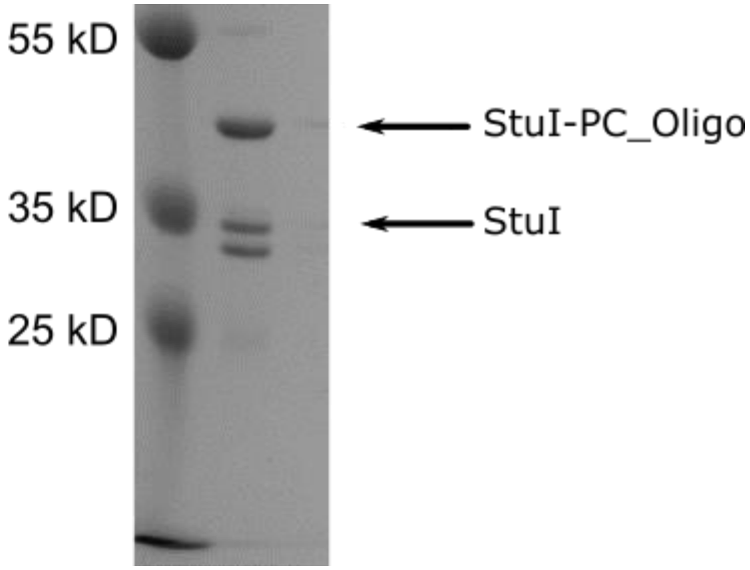

Figure S3. Sample purity of the StuI-PC_Oligo sample. SDS-PAGE gel of StuI-PC_oligo sample. Unmodified StuI enzyme was always co-eluted with the StuI-PC_oligo species. Furthermore, a contaminant of lower molecular weight than StuI co-eluted as well with the enzyme during the previous purification steps. We assigned this contamination to a partially degraded form of the enzyme, as it always co-eluted with the protein after different chromatographic steps (including Nickel affinity purification, cationic exchange, anionic exchange, and hydrophobic interaction chromatography). We interpreted the consistent co-elution of the different species to the formation of oligomers, which is consistent with the oligomeric nature of type II restriction enzymes.
